# Nrf2 Signaling Pathway Mediates the Protective Effects of Daphnetin Against D-Galactose Induced-Premature Ovarian Failure

**DOI:** 10.3389/fphar.2022.810524

**Published:** 2022-01-28

**Authors:** Mengwen Zhang, Xiaowei Yu, Danjie Li, Ning Ma, Zhentong Wei, Xinxin Ci, Songling Zhang

**Affiliations:** ^1^ Department of Obstetrics and Gynecology, The First Hospital of Jilin University, Changchun, China; ^2^ Institute of Translational Medicine, The First Hospital of Jilin University, Changchun, China

**Keywords:** premature ovarian failure, Nrf2, NLRP3, Txnip, daphentin

## Abstract

Oxidative damage can lead to severe ovarian dysfunctions and even premature ovarian failure. Nrf2, a significant transcription factor that regulates the oxidative stress response of cells, declines with age. Daphnetin, as a kind of natural Chinese herbal medicine, can activate Nrf2 and further promote the antioxidant defense of cells. However, whether Daphnetin treatment can protect ovary from premature ovarian failure and the specific mechanism involved are not understood. This study aimed to investigate the protective function of Daphnetin against the ovarian aging induced by D-galactose in wild-type and Nrf2^−/−^ mice. Female C57BL/6 mice with Wild-type and Nrf2^−/−^ were divided into five groups separately and the premature ovarian failure model were established by D-galactose and then Daphnetin and VE were given for treatment. After 42 days, ovaries tissue and serum were collected for biochemical determination, H&E staining, Immunohistochemical staining and western blot analysis. In the WT-POF group, ovarian function was broke, and the expression of the ovarian senescence-associated protein P16 and the level of oxidative stress were significantly increased, while the expression of the anti-senescence protein klotho was significantly decreased. In addition, the expression of Nrf2 and the antioxidases GCLC, HO-1 and NQO1 were decreased, but TXNIP and NLRP3 were significantly increased. Furthermore, the characteristics of premature ovarian failure were more significant in Nrf2 knockout mice than in wild-type mice, especially the expression of NLRP3 and TXNIP. Moreover, daphnetin, an Nrf2 activator, rescued d-gal-induced POF in a dose-dependent manner, while the protective effect was weakened or even lost in Nrf2 knockout mice. Our results suggested that daphnetin is likely to be a candidate drug for premature ovarian failure treatment and it is mostly possible referred to the molecular mechanism of increasing Nrf2 expression and inhibiting NLRP3 activation in the ovarian aging process.

## Introduction

Premature ovarian failure (POF), the current term for ovarian dysfunction called premature ovarian insufficiency (POI), occurs in women under 40 years of age whose follicle-stimulating hormone (FSH) level is > 40 IU/ml and is accompanied by secondary amenorrhea, infertility and low estrogen symptoms (Perry et al., 2013; Hewlett and Mahalingaiah, 2015). Epidemiological surveys have shown that the prevalence of POF in the population is approximately 1–3%, and in amenorrheic patients, it is approximately 2–10% (Rafique et al., 2012). In recent years, the incidence of POF has been increasing year by year because of factors such as the environment and the increase in women’s work intensity. Clinically, POF is a highly heterogeneous female disease with extremely complex etiology, and risk factors include genetics, autoimmune, metabolic disorders and iatrogenic diseases, which contribute to the difficulty of treatment (Qin et al., 2015; Kirshenbaum and Orvieto, 2019; Christianson et al., 2020). Hormone replacement therapy (HRT) is now being abandoned for some female patients because of the potential risk of endometrial, breast cancer and thrombotic diseases (Deady, 2004). The ovarian stem cell treatment method is still under investigation (Wang et al., 2013).

Previous studies have indicated that oxidative damage in the ovary can lead to severe ovarian dysfunctions (Pandey et al., 2018). As age increases, the increase in free radical production in the ovaries and the reduction in antioxidants diminish the capability of the ovaries to scavenge free radicals (Bandyopadhyay et al., 2003). Nuclear factor erythroid-2 related factor 2 (Nrf2) is a critical transcription factor that regulates the oxidative stress reaction of cells. Meanwhile, it also acts as a central regulator to maintain the redox equilibrium of cells and the body. Nrf2 can protect cells from damage caused by reactive oxygen species (ROS) and keep them in a stable state by regulating the expression of complex downstream antioxidant enzymes, such as glutamate cysteine ligase catalytic (GCLC), NAD(P)H dehydrogenase quinine 1 (NQO1) and heme oxygenase-1 (HO-1) (Latella, 2018; Tonelli et al., 2018). Studies have shown that Nrf2 knockouts can contribute to an increase in the prevalence of age-related diseases, such as cancers, kidney damage, pulmonary vascular remodeling, liver damage and coronary heart disease, and could be associated with reduced life expectancy and premature aging (Volonte et al., 2013; Duarte et al., 2017). In addition, overexpression of senescence-associated protein P16 protein can induce premature senescence of cells, while the anti-senescence protein klotho inhibits ovarian and endothelial cell senescence by activating the Nrf2 pathway (Romero et al., 2019; Chen et al., 2021). A number of recent preclinical reports have shown that the application of antioxidants such as Nrf2 activators can scavenge excessive ROS and decrease inflammatory responses by suppressing NLRP3 inflammasome activation (Wang et al., 2016; Xu et al., 2019). Moreover, studies have shown that inhibition of NLR family pyrin domain containing 3 (NLRP3) inflammasome activation prevents ovarian aging (Navarro-Pando et al., 2021). Currently, the Nrf2-related oxidative stress theory about the mechanism underlying POF has been discussed extensively, but the specific mechanism is not yet clear (Yan et al., 2018; Li et al., 2019).

It is well known that cellular damage caused by ROS continuously generated during cell metabolism is one of the fundamental causes of senescence in the body, and maintaining appropriate antioxidants and free radical scavengers can prolong life span (Lee and Wei, 2001). Treatment of mice with D-gal can lead to excessive production of ROS and the accumulation of advanced glycation end products (AGEs) in the ovaries, damaging ovarian physiological function. Because the accelerated aging process in the aging model induced by D-gal is highly similar to the process that occurs during human aging, researchers have used this approach to establish a POF model in mice (Song et al., 1999) (Semba et al., 2010). However, the role and mechanism of the Nrf2 signaling pathway in D-gal-induced POF have not been clarified. Daphnetin (Daph) acts as an activator of Nrf2, enhancing the antioxidant defenses of liver, kidney and heart cells through activating the Nrf2 pathway (Fan et al., 2020; Lv et al., 2020). In this study, we detected that the Nrf2/TXNIP/NLRP3 axis was regulated in a D-gal-induced POF model. Furthermore, activation of Nrf2 and inhibition of NLRP3 by Daph significantly protected against D-gal-induced POF.

## Materials and Methods

### Reagents

D-gal (D-galactose, C_6_H_12_O_6_) was acquired from Sigma-Aldrich Chemical Co. (Switzerland), assay: ≥99%. Daphnetin (C_9_H_6_O_2_) was obtained from Sigma-Aldrich Chemical Co. (United States), assay: ≥97%. VE (Vitamin E, C_25_H_5_O_2_) was obtained from Dalian Meilun Biotechnology (China), assay: ≥98%. Sodium chloride injection was acquired from Jilin Dubang Pharmaceutical Co., Ltd. Jilin, China. Ethanol absolute was obtained from Tianjin Xinbote Chemical Co., Ltd. Tianjin, China.

### Animals and Treatment

Female C57BL/6 mice aged 6–8 weeks were purchased from Vital River Laboratory Animal Technology Co., Ltd. (Beijing, China). Nrf2-deficient mice (Nrf2^−/−^) were supplied by the Jackson Laboratory (Bar Harbor, ME, United States). Food and water were provided via free choice. Firstly, mice were randomly divided into 4 independent groups (5 mice per group): saline, D-gal: 200 mg/kg/d, 400 mg/kg/d and 600 mg/kg/d. All mice were subcutaneous injected daily for 42 days to determine the appropriate D-gal concentration for the POF induction. Then, mice were randomly divided into the following five groups (5 mice per group, and every group mice were injected daily for 42 days): Negative control group: subcutaneous injection of saline; D-gal group: subcutaneous injection of D-gal 600 mg/kg/d; Daph group (low- or high-does): intraperitoneal injection of Daph (15 or 30 mg/kg/d) after subcutaneous injection of D-gal; Positive control group: oral administration of Vitamin E (VE) (30 mg/kg/d) after subcutaneous injection of D-gal. Female Nrf2 ^−/−^ mice were also divided into five groups following the above method. All animals involved in the study were approved by the Animal Health and Research Ethics Committee of Jilin University.

### Oestrous Cycles

Oestrous cycles were monitored through vaginal smear after D-gal treatment for consecutive 12 days to evaluate the oestrous cycle stages of the mice (5 mice per group). Vaginal lavage sample were diluted with sodium chloride injection at an appropriate concentration. Then the cells were distributed evenly on a clean slide through Centrifugal smear dyeing machin (CYTOPRO, Cytoprp 7,621), fixed in Ethanol absolute for 10 s and stained with Wright-Giemsa Stain (Baso Diagnostics Inc. Zhuhai, China). Vaginal cells were analysed by light microscopy in terms of morphological criteria. We assessed the length of each cycle as the length of time between two consecutive occurrences of oestrus.

### Serum Preparation and Hormones and Oxidative Stress Level Measurement

Blood samples were collected by retro-orbital bleeding while mice were during the dioestrus, and then, samples were allowed to clot at room temperature for 60 min. At last, the samples were centrifuged at 2,500 rpm for 15 min to harvest serum, which were stored at −80°C. The commercially available ELISA kits FSH (ml001876) and E2 (ml063198) were purchased from Shanghai Enzyme-linked Biotechnology Co., Ltd. and were used to measure the serum follicle-stimulating hormone (FSH) and estradiol (E2) levels of samples. The levels of oxidative stress in ovaries, including the total superoxide dismutase (SOD) activity and L-Glutathione (GSH) level, were measured by using commercially available kits for SOD (A001-3-2) and GSH (A005-1-2) (Jiancheng Bioengineering Institute, Nanjing, China). All steps were performed according to the manufacturer’s instructions.

### Hematoxylin and Eosin Staining and Follicle Number Counting

The right ovaries (5 mice per group) were embedded in paraffin after being fixed in 4% paraformaldehyde overnight. The tissues were cut into 4-μm-thick sections for H&E staining. Only follicles with a visible oocyte nucleus were counted to avoid repeat counting. The follicle classification system was used: primordial follicles were identified as those containing a primary oocyte in the center and surrounded by a monolayer of flattened granulosa cells; primary follicles were characterised by the presence of an enlarged oocyte surrounded by one layer of columnar granulosa cells and with red-stained zona pellucida between the egg and the oocyte; secondary follicles containing more than one layer of columnar granulosa cells and no visible antrum surrounding the oocyte; antral follicles have follicular fluid or a single large antral space; and atretic follicles were identified as those entring into a degenerative process and the follicles were replaced by fibrous connective tissue.

### Immunohistochemical Staining

The paraffin sections were sequentially prepared according to the following instructions: dewaxing of the sections, antigen retrieval, blocking with endogenous peroxidase, blocking with 10% normal rabbit serum and incubation at room temperature for 30 min. The primary antibodies anti-p16 (380,928) (Zhengneng Biotechnology), anti-klotho 382,164) (Zhengneng Biotechnology), anti-TXNIP (ab188865) (Abcam) and anti-NLRP3 (19771-1-AP) (Proteintech) were added to the slices at appropriate dilution concentrations and incubated at 4°C overnight. They were then rinsed with PBS for 5 min 3 times and incubated with biotin-labeled secondary antibody for 30 min at 37°C. And then, they were rinsed with PBS for 5 min 3 times again. The SP Rabbit & Mouse HRP Kit (DAB) was developed for 10 min, rinsed thoroughly with PBS, counterstained, dehydrated, transparent, and mounted. Tissue sections were observed and photographed with a microscope, and Image-Pro Plus 6.0 software was used for semi-quantification. Staining was quantified by measuring the immunoreactive area (IA) in μm^2^ and the integrated optical density (IOD). The staining intensity (SI) for each image was calculated as SI = IOD/IA.

### Western Blot Analysis

Protein expression was analyzed by western blot as described previously. Equal amounts of ovarian protein lysate from each group were added to the lanes of gels for electrophoresis and then transferred to a polyvinylidene fluoride membrane. Then, the membrane was blocked with 5% nonfat milk at room temperature for 1 h, eluted with PBST and incubated with primary antibodies against Nrf2 (16396-1-AP) (Proteintech), GCLC (12601-1-AP) (Proteintech), NQO1 (ab80588) (Abcam), HO-1 (ab189491) (Abcam), p16 (380,928) (Zhengneng Biotechnology) and klotho 382,164) (Zhengneng Biotechnology) at appropriate dilutions overnight at 4°C. The membranes were washed 3 times using PBST and then incubated in secondary antibody for 1 h. The membranes were detected by ECL and analyzed by Image Lab 4.0.1.

### Statistical Analysis

All the studies and the results’ analyses were repeated at least 3 times. The data are shown as the mean ± SEMs and were analyzed using SPSS 19.0 (IBM). The experimental data were compared by one-way analysis of variance (ANOVA). A *p* value <0.05 was considered statistically significant. *p < 0.05 and **p < 0.01 compared with the negative control group; ^#^ *p* < 0.05 and ^##^ *p* < 0.01 compared with the D-gal (600 mg/kg/d) group.

## Results

### High-Dose D-Gal (600 mg/kg/day)-Induced POF Mouse Model

**FIGURE 1:**
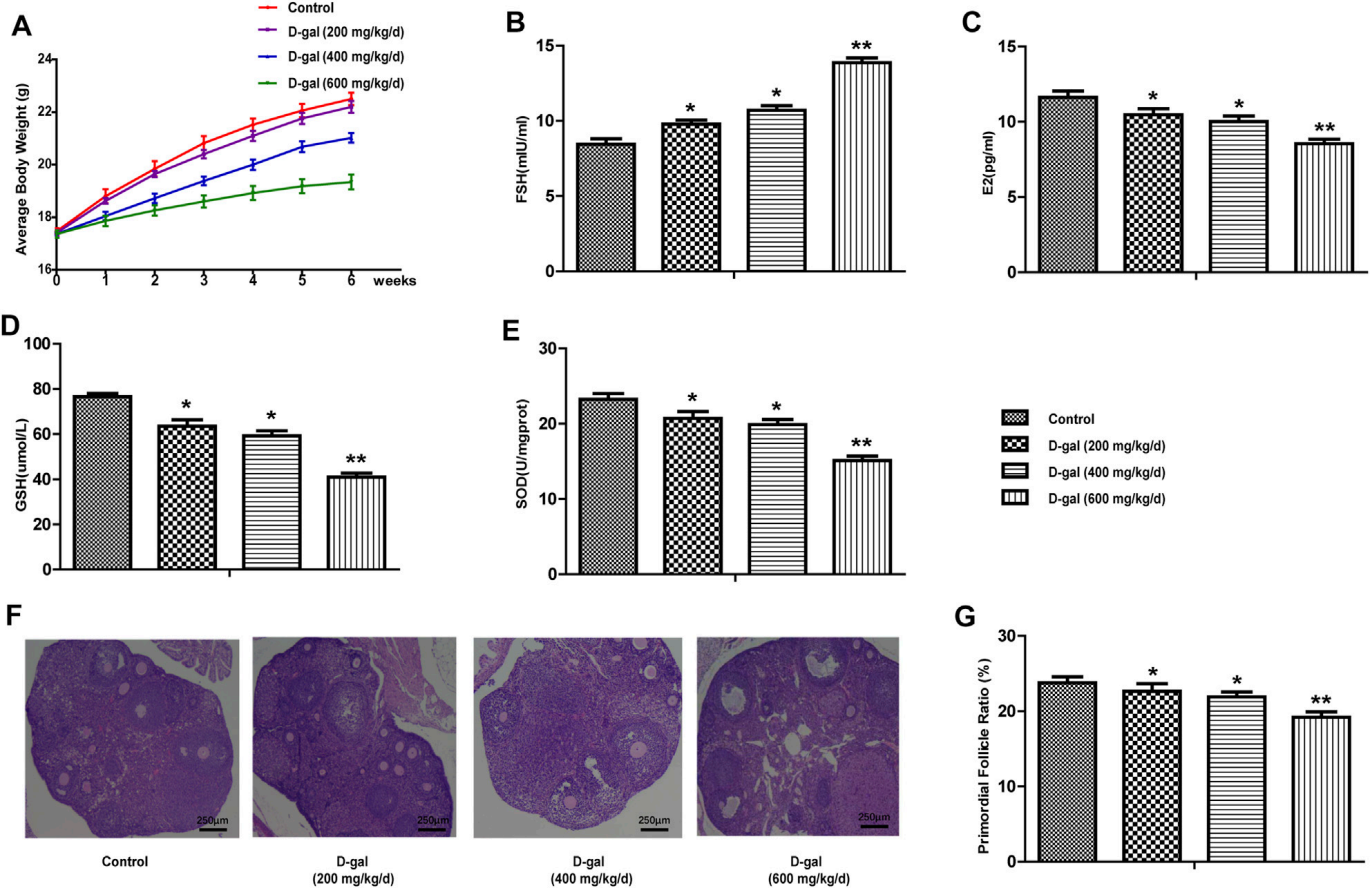
Establish a mouse model of POF. **(A)** The body weight changes of mice in control and D-gal group (low, middle and high dose) were recorded during the process of inducing the POF model. **(B,C)** The serum FSH and E2 levels were tested in four groups. **(D,E)** The levels of GSH and SOD in the ovarian tissues were tested. **(F,G)** The proportion of primordial follicle. All data are shown as means ± SEMs, *n* = 5. Statistical significance: *p < 0.05, **p < 0.01.

In this study, we first chose 200, 400 and 600 mg/kg/d D-gal to induce ovarian aging in mice. As shown in Figure 1A, the mice in the high-dose D-gal group gradually showed signs of decreased activity, reduced food intake, depilation, poor mental state, and slow weight gain; this phenomenon was not observed in the other three D-gal groups. In Figure 1B, compared with the control group, the serum FSH was significantly increased in the 600 mg/kg/d-dose D-gal group. Moreover, serum E2 levels, the expression levels of GSH and SOD in the ovarian tissues showed the opposite trend compared to FSH (Figures 1C–E). In addition, Figures 1F,G indicate that the proportion of primordial follicles in the D-gal group (high-dose group) was more significantly reduced. Therefore, in this study, we selected 600 mg/kg/d of D-gal as the appropriate concentration to establish a mouse model of POF.

### Nrf2 Knockout Significantly Damages the Ovarian Function and Antioxidant Capacity of POF Mice

**FIGURE 2:**
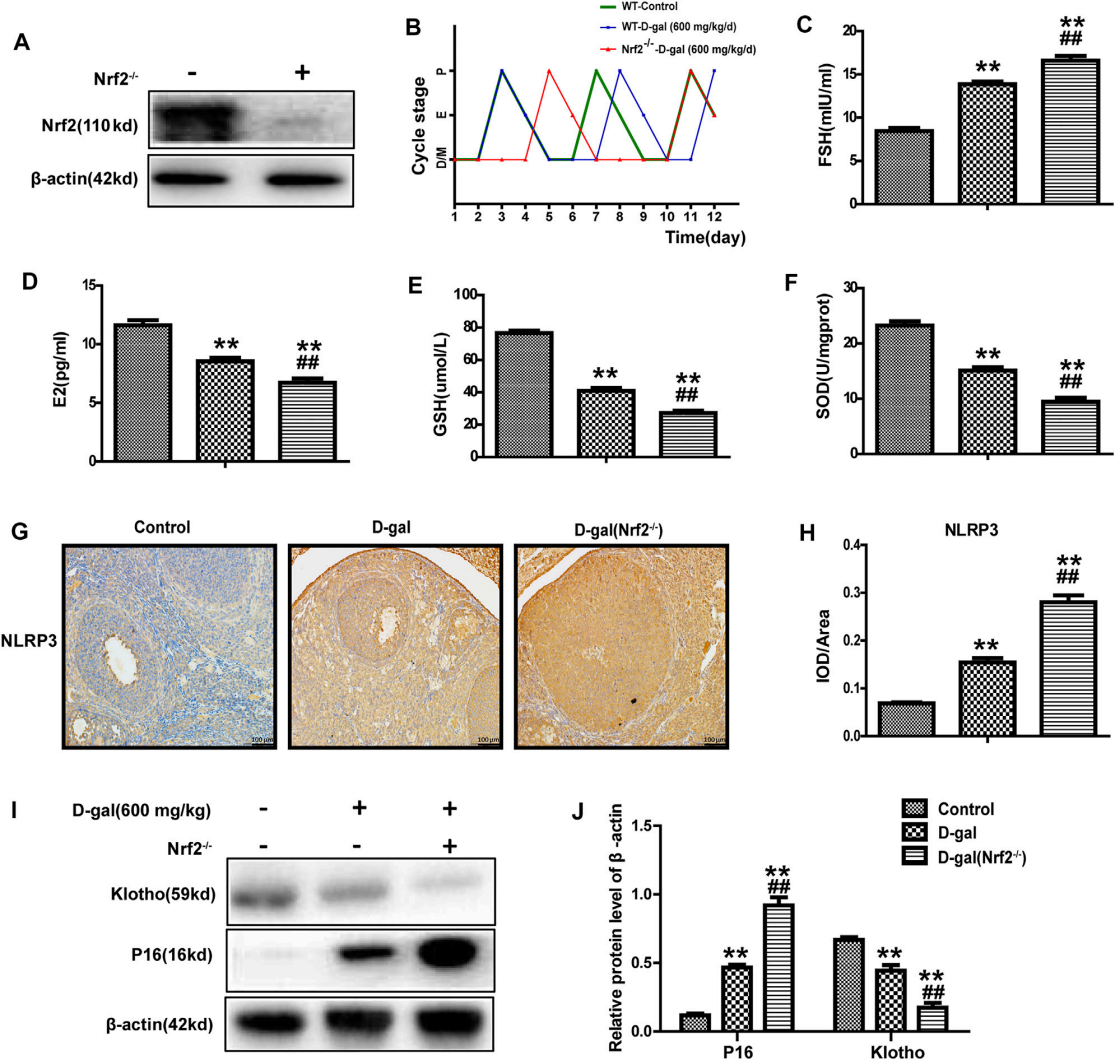
Nrf2 knockout significantly damaged the ovarian function and antioxidant capacity of POF mice. **(A)** Immunoblotting analysis of Nrf2 protein. **(B)** Using vaginal cytology evaluating the oestrous cycle and days spent in pro-oestrus (P), oestrus (E), metoestrus (M), and dioestrus (D) over the course of 12 consecutive days. **(C,D)** The serum FSH and E2 levels were tested. **(E,F)** The levels of GSH and SOD in ovarian tissues. **(G,H)** NLRP3 protein expression was analyzed after immunohistochemistry. **(I,J)** The level of P16 and klotho were analyzed. The data in B-H are shown as means ± SEMs, *n* = 5. The data in A, I-J are shown as means ± SEMs, *n* = 3. Statistical significance: **p < 0.01, ^##^ *p* < 0.01.

Studies have shown that Nrf2 plays a crucial role in premature ovarian failure. Next, as mentioned above, we chose D-gal (600 mg/kg/day) to construct a POF model *via* subcutaneous injection in wild-type and Nrf2^−/−^ mice. Immunoblotting identified the genotypes of Nrf2^−/−^ mice (Figure 2A). Oestrous cycles were gained through a daily vaginal smear. The results showed that the control group exhibited regular oestrous cycles about 4 days, and the using of D-gal could lengthen the cycle from 4 to 5 days; however, this phenomenon was more significant in Nrf2^−/−^ mice with an obviously increased length of the dioestrus period (Figure 2B). Compared with the wild-type mice, D-gal more significantly regulated the levels of serum FSH and E2 in the Nrf2^−/−^ mice (Figures 2C,D). Figures 2E,F show that GSH and SOD levels were lower in the Nrf2^−/−^ group. Moreover, D-gal-induced NLRP3 and P16 were much higher in Nrf2^−/−^ mice, while klotho was much lower (Figures 2G–J). These results indicated that Nrf2 plays significant effect in ovarian function of POF mice.

### Daph Protects Ovarian Function and Follicular Development in POF Mice

**FIGURE 3:**
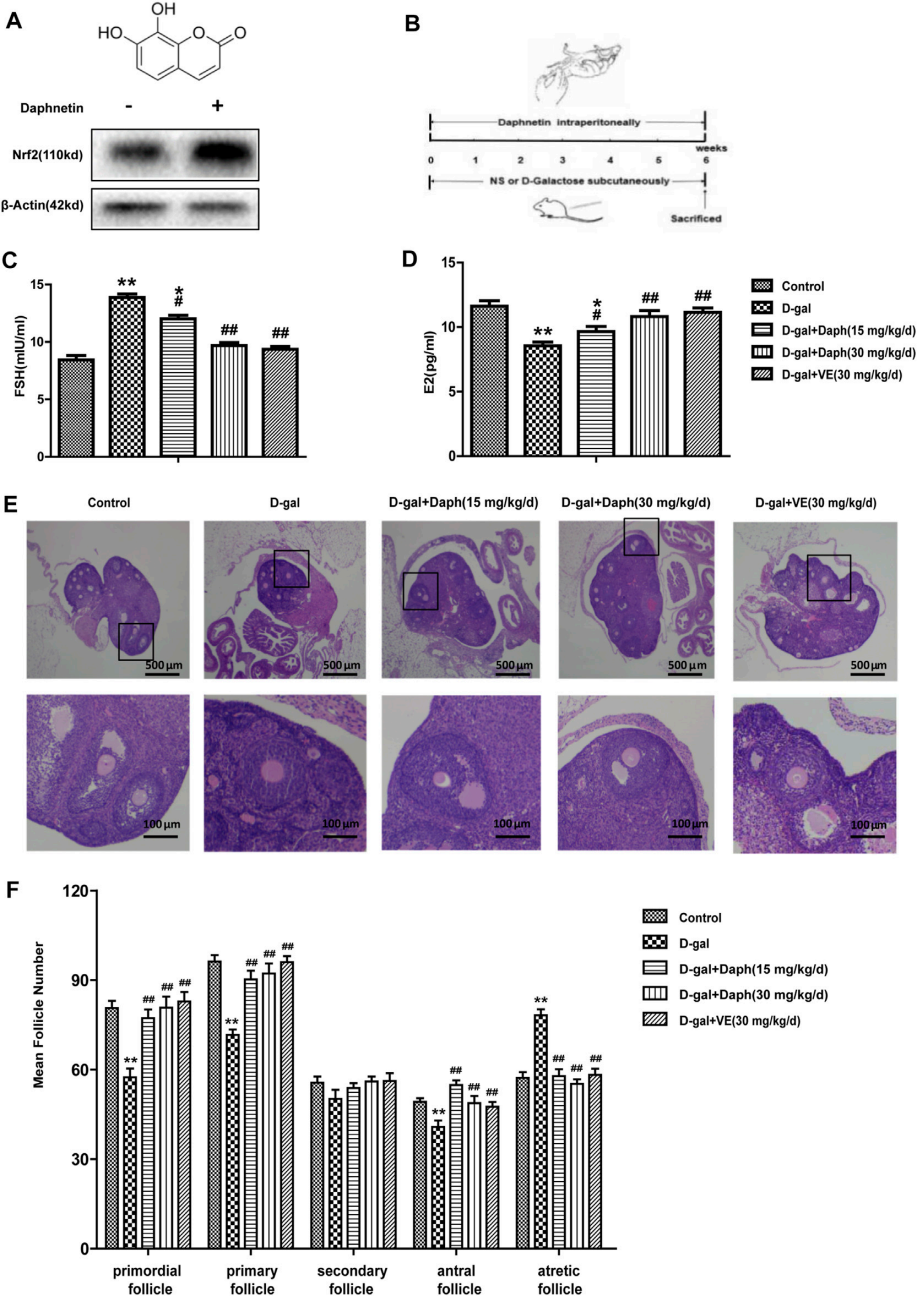
Protective effects of Daph on the ovarian function and follicular development. **(A)** The structure of Daph monomer and immunoblotting analysis of Nrf2 protein expression after Daph was administrated. **(B)** Construct mice model according to the method as shown. **(C,D)** Serum FSH and E2 were tested in the control, D-gal, Daph and VE groups. **(E,F)** Follicles in five groups were observed after H&E staining and the numbers of follicles at different stages of maturation were summarized. The data in C-F are shown as means ± SEMs, *n* = 5. The data in A are shown as means ± SEMs, *n* = 3. Statistical significance: *p < 0.05, **p < 0.01, ^#^ *p* < 0.05, ^##^ *p* < 0.01.

Previous results indicated that Nrf2 has a vital effect on POF. Next, we further explored the protective effect of Daph, a Nrf2 activator, on D-gal-induced POF. As shown in Figure 3A, Daph increased Nrf2 expression in ovaries. Furthermore, Daph (15 mg/kg/d) significantly decreased serum FSH levels and increased E2 levels, while its 30 mg/kg/d dose and VE almost restored FSH and E2 to the levels of the control group, indicating that Daph significantly rescued the ovarian function injury caused by D-gal (Figures 3C,D). After H&E staining, the follicles were classified and counted based on the methods previously described (Figure 3E). The results showed that D-gal treatment reduced the proportion of primordial follicles, primary follicles and antral follicles, and increased the proportion of atretic follicles (Figure 3F). In contrast, the follicle counts of the 15 mg/kg/d-dose Daph treatment group increased significantly at different stages of maturation, and the 30 mg/kg/d-dose Daph and VE almost fully demonstrated its protective effect on follicle development, although secondary follicles have not obvious change (Figure 3F). The above results showed that Daph can protect ovarian function and follicular development in POF mice in a concentration dependent-manner.

### Effects of Daph on P16 and Klotho Protein Expression

**FIGURE 4:**
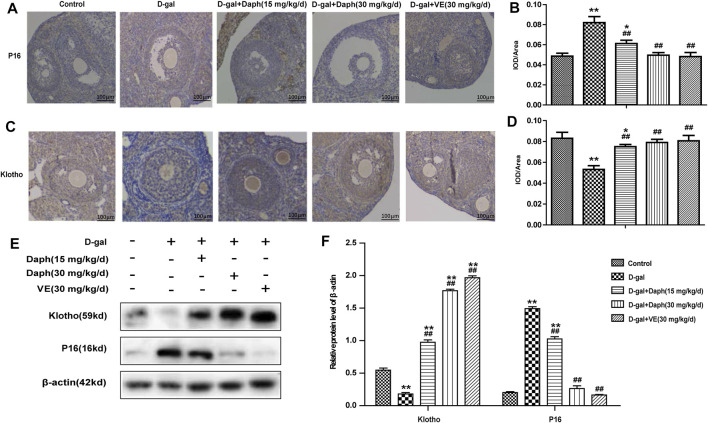
Effects of Daph on p16 and klotho expression. **(A,C)** The IHC staining showed cellular locations of p16 and klotho proteins in different groups. **(B,D)** The p16 and klotho expressions were quantitatively analyzed. **(E,F)** Expressions of p16 and klotho were verified by western blot. The data in **(A–D)** are shown as means ± SEMs, *n* = 5. The data in **(E–F)** are shown as means ± SEMs, *n* = 3. Statistical significance: *p < 0.05, **p < 0.01, ^##^ *p* < 0.01.

Next, immunohistochemistry was performed to examine the expression of p16 and klotho in ovarian tissues. P16 was mostly located in follicular granulosa cells and oocytes. D-gal treatment led to a noteworthy increase in p16 protein expression compared with the control group, which was reversed by Daph and VE treatment (Figures 4A,B). Studies have shown that klotho participates in the development of follicles as an anti-senescence protein. The level of klotho protein were significantly lower in the D-gal group but partially increased in the Daph and VE group (Figures 4C,D). Consistent with the immunohistochemistry results, western blotting also confirmed the same tendency (Figures 4E,F), which indicated that Daph has a protective effect on cell senescence in a POF mouse model.

### Effect of Daph on Oxidative Stress-Related Pathways in the Ovary

**FIGURE 5:**
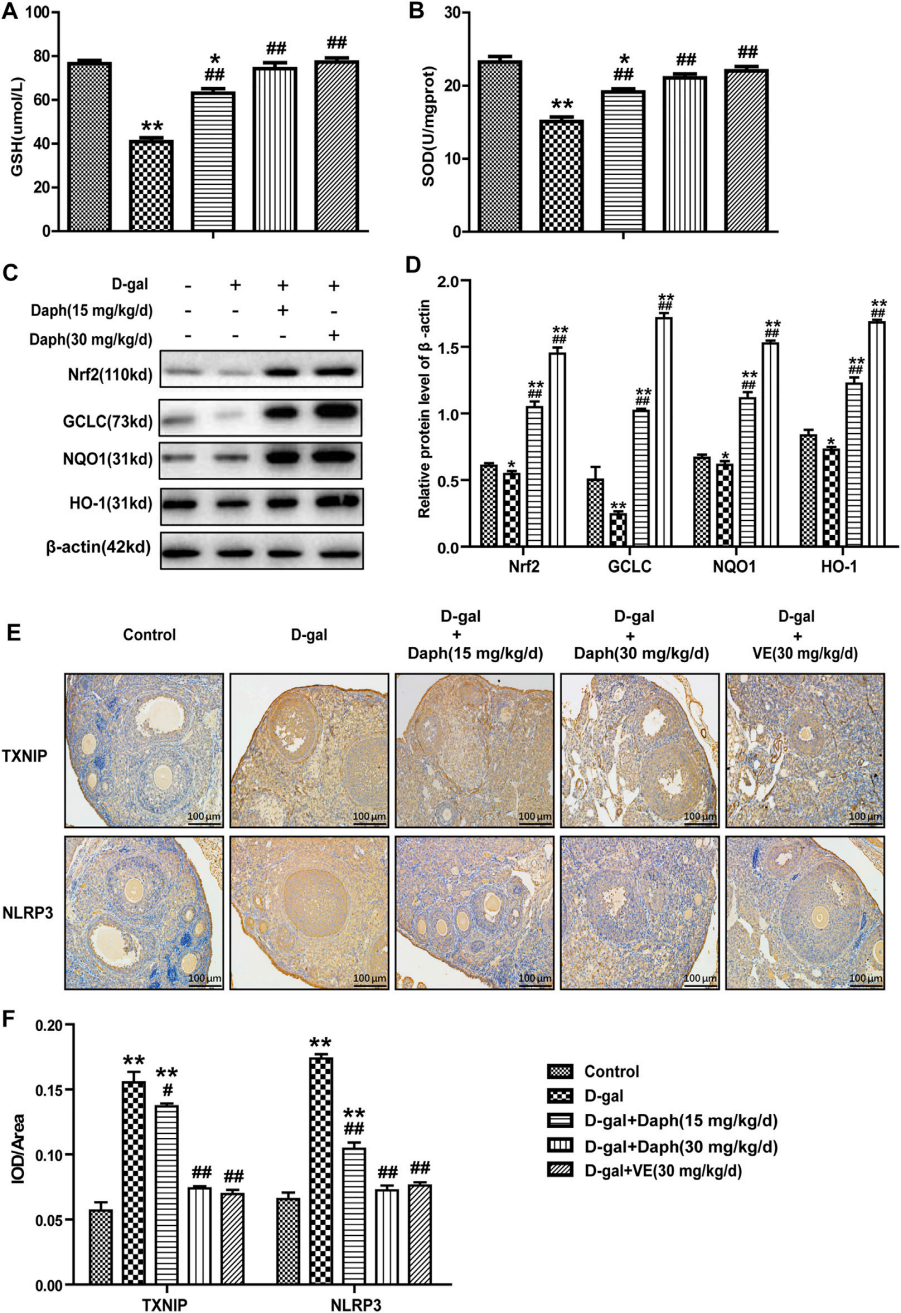
Effect of Daph on oxidative stress-related pathways in the ovary. **(A,B)** The total SOD enzyme activity and the GSH level were measured in ovarian tissues. **(C)** The levels of Nrf2, GCLC, NQO1 and HO-1 protein expression in ovarian tissues were observed via Western blotting. **(D)** The protein expression levels were quantitatively analyzed. **(E,F)** TXNIP and NLRP3 protein expression in the five groups using IHC staining. All data are shown as means ± SEMs, *n* = 5, besides **(C,D)** which data are shown as means ± SEMs, *n* = 3. Statistical significance: *p < 0.05, **p < 0.01, ^#^ *p* < 0.05, ^##^ *p* < 0.01.

Compared to the control group, the total SOD enzyme activity and antioxidant GSH were significantly reduced by D-gal treatment in ovarian tissues but were partially rescued by Daph and VE treatment (Figures 5A,B). Next, we further researched the possible mechanisms involved in the effects of Daph on D-gal-induced POF. As shown in Figures 5C,D, expression of Nrf2 and its downstream antioxidant enzymes GCLC, HO-1 and NQO1 were markedly decreased in the D-gal group but upregulated by Daph treatment in a dose-dependent manner. As mentioned above, the NLRP3 inflammasome has an important effect on ovarian aging, and high TXNIP protein expression indicates oxidative damage to cells. As shown in Figures 5E,F, TXNIP and NLRP3 protein expressions were markedly increased in the D-gal group versus the control group but were significantly decreased by Daph and VE treatment.

### Knockout of Nrf2 Abolished the Protective Capability of Daph for Ovarian Function and Follicular Development

**FIGURE 6:**
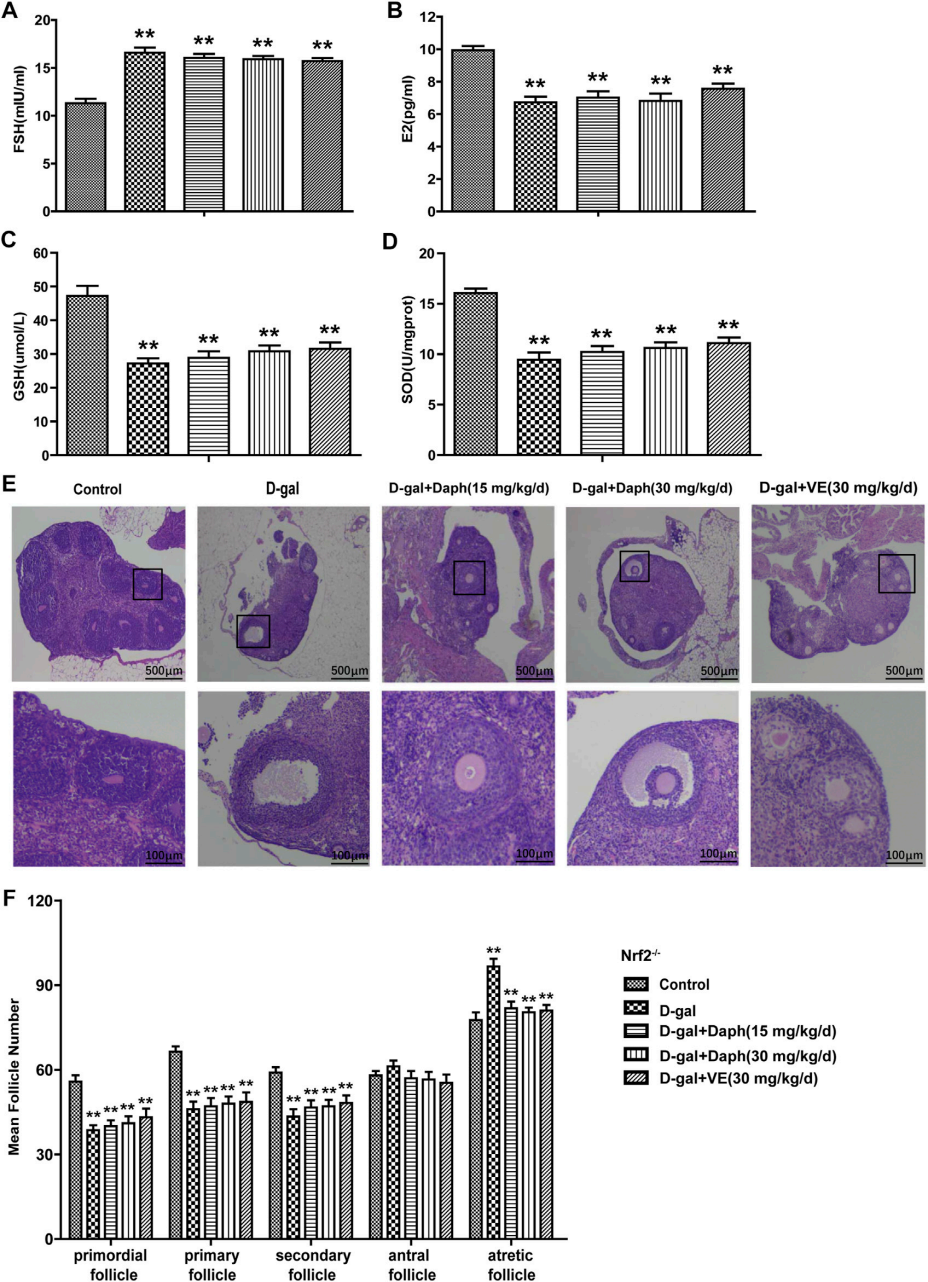
Knockout of Nrf2 abolished the protective capability of Daph for ovarian function and follicular development. **(A,B)** The serum FSH and E2 levels were tested in the five groups in Nrf2^−/−^ mice. **(C,D)** GSH and SOD expression in ovarian tissues was tested. **(E)** Follicles were observed after H&E staining. **(F)** Summarizing the numbers of follicles at different developmental stages. All data are shown as means ± SEMs, *n* = 5. Statistical significance: **p < 0.01.

As shown in Figures 6A,B, Daph could not rescue serum hormones regulation induced by D-gal in Nrf2 knockout mice, including FSH upregulation and E2 downregulation. Meanwhile, GSH and SOD depletion induced by D-Gal could not be reversed by daphnetin treatment (Figures 6C,D ). Furthermore, compared with the control group, Daph did not increase the proportion of primordial or primary follicles or decrease the proportion of atretic follicles after D-gal treatment in Nrf2^−/−^ mice (Figures 6E,F). These results demonstrated that the protective capability of Daph for ovarian function and follicular development was abolished in Nrf2 knockout mice.

### Knockout of Nrf2 Abolished the Regulatory Capability of Daph for Ovarian Senescence-Associated Protein Expression

**FIGURE 7:**
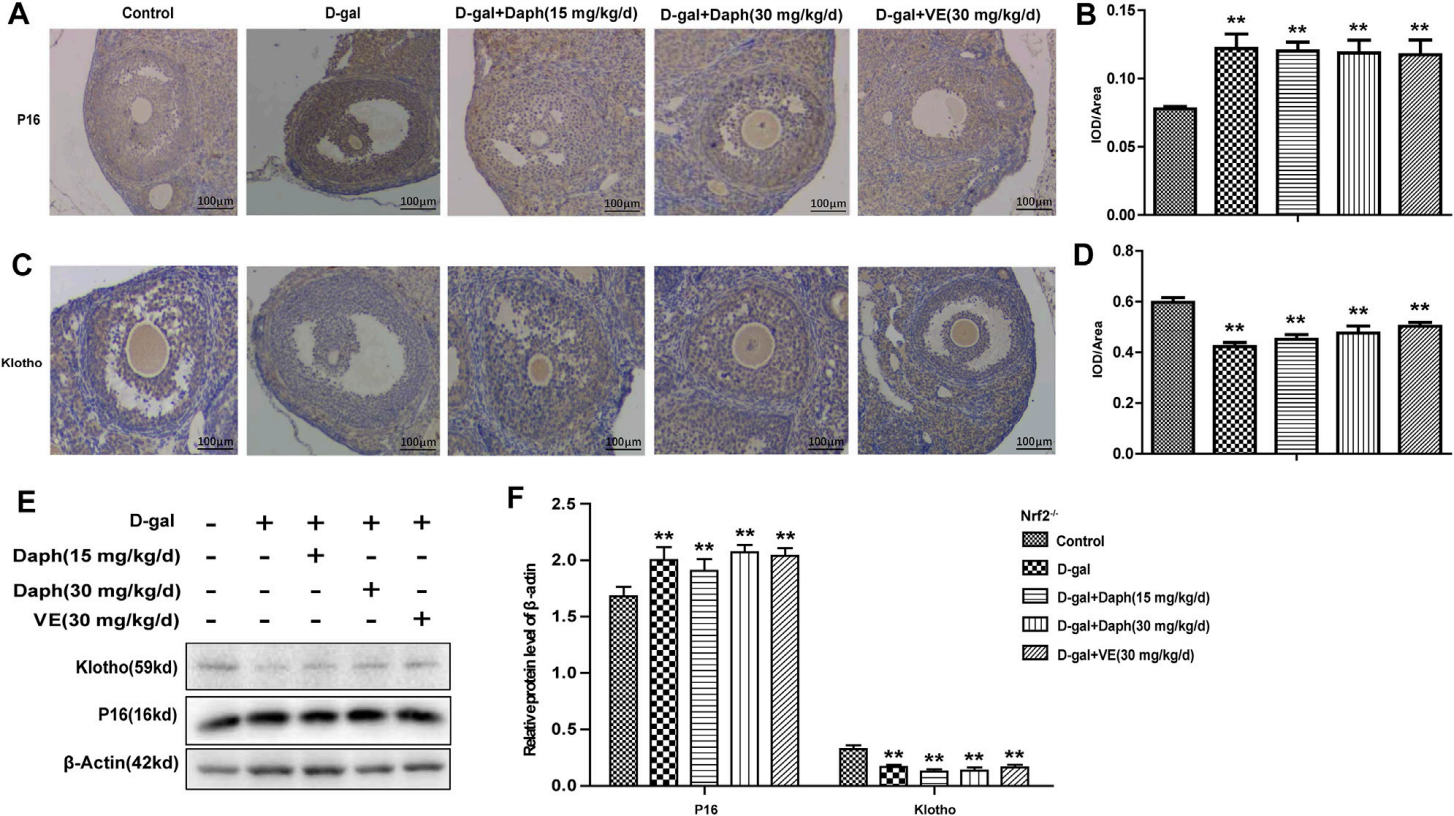
Knockout of Nrf2 abolished the regulatory capability of Daph for ovarian senescence-associated protein expression. **(A–D)** The IHC staining showed the p16 and klotho expressions in the five groups in Nrf2^−/−^ mice. **(E,F)** Expressions of p16 and klotho were measured through western blot. The data in **(A–D)** are shown as means ± SEMs, *n* = 5. The data in **(E,F)** are shown as means ± SEMs, *n* = 3. Statistical significance: **p < 0.01.

In Figures 7A–D, compared to the control group, Daph and VE could not recover ovarian senescence-associated protein expression from increased P16 or decreased klotho by D-gal in Nrf2^−/−^ mice. Consistent with these immunohistochemistry results, western blot analysis also verified this trend (Figures 7E,F). These results indicated that Daph lost the capability of recovering ovarian senescence-associated protein expression after Nrf2 knockout.

### Knockout of Nrf2 Abolished the Adjustive Power of Daph on Oxidative Stress-Related Pathways

**FIGURE 8:**
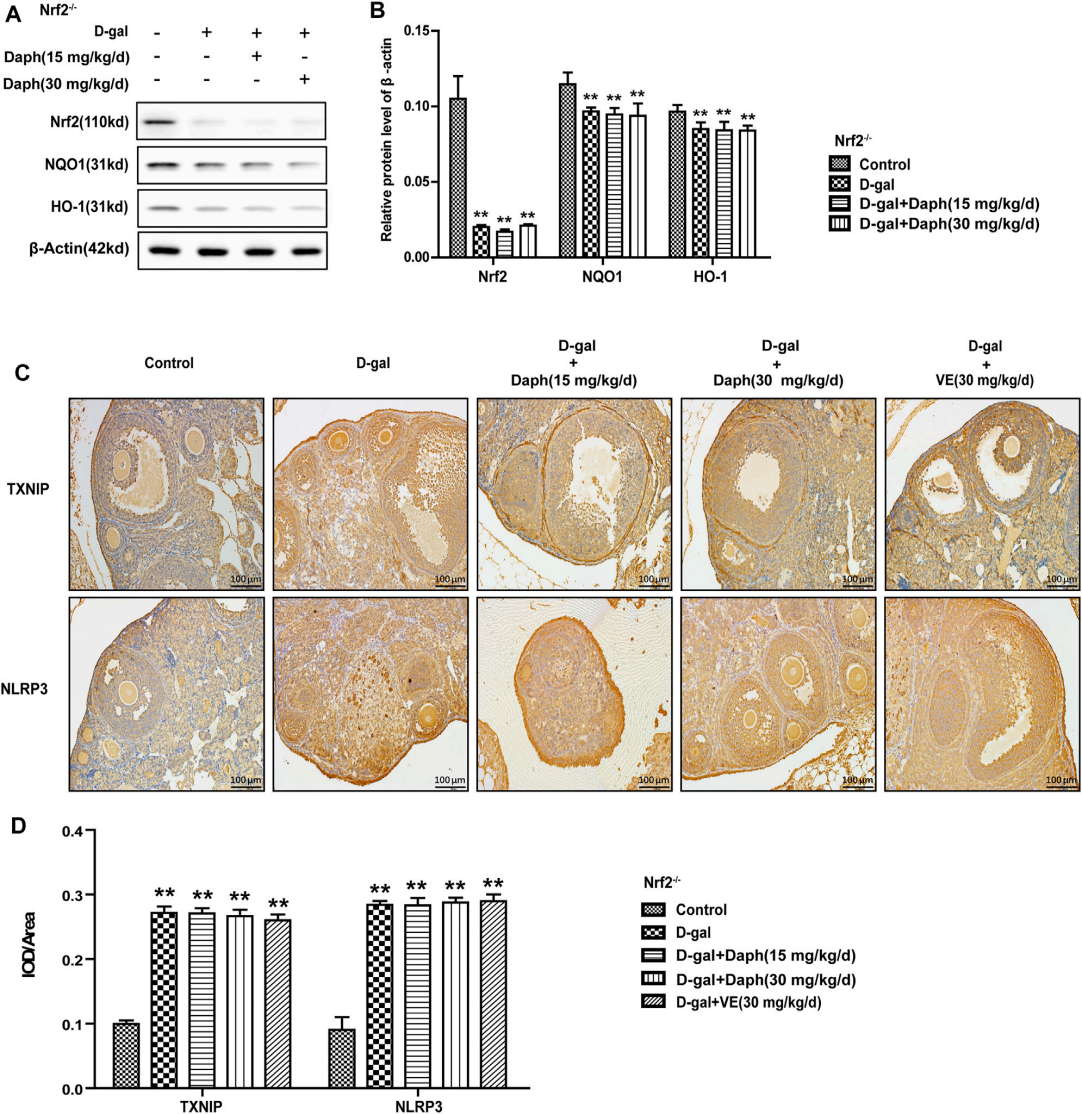
Knockout of Nrf2 abolished the adjustive power of Daph on oxidative stress-related pathways. **(A,B)** The Nrf2, NQO1 and HO-1 protein expression levels in ovarian tissues were tested by Western blotting. **(C,D)** TXNIP and NLPR3 protein expression level analysis *via* immunohistochemistry. The data in **(A,B)** are shown as means ± SEMs, *n* = 3. The data in **(C,D)** are shown as means ± SEMs, *n* = 5. Statistical significance: **p < 0.01.

In Nrf2^−/−^ mice, the levels of Nrf2 and its antioxidases (NQO1 and HO-1) were significantly decreased, while Daph treatment did not save this tendency (Figures 8A,B). Daph and VE did not reverse the increased expression of the oxidative stress-related factors TXNIP or NLRP3 induced by D-gal in Nrf2^−/−^ mice (Figures 8C,D).

## Discussion

For human body, ROS are the critical factors in aging, and the decreasing of Nrf2 level is also a key contributor. Mechanically, Nrf2 can upregulate associated genes combating oxidative stress, and the loss of Nrf2 can accelerate the aging process (Schmidlin et al., 2019). However, the function and the specific mechanism of Nrf2 in the POF are not known and which are the points of this study. Daphnetin, an activator of Nrf2, is derived from a natural Chinese herbal medicine and enhances the antioxidant defense of liver, kidney and heart cells via activating the Nrf2 pathway (Zhang et al., 2020) (Lv et al., 2020). However, its function in ovary aging remains unclear, which will be further clarified in this study.

Previous researches have shown that using D-gal could contribute to an increase in ROS and AGE levels, an increase in granulosa cell apoptosis, and impaired follicular development. D-gal induces oxidative stress in the body, and the toxicity of galactose reduces the biological activity of FSH and inhibits the production of E2 by granulosa cells (Song et al., 1999; Semba et al., 2010). In terms of treatment, HRT is one of the recommended methods for the treatment of POF, but long-term estrogen therapy may increase the chance of endometrial cancer, breast cancer and thrombotic diseases in patients (Sullivan et al., 2016). In recent years, stem cell therapy for POF has also received increasing attention (Li et al., 2017; Mohamed et al., 2018). Despite these advances, the prevention and treatment of POF are still challenging because of the limitations of existing methods. In short, exploring the pathogenesis and available therapies are critical for preventing and treating POF. Our study successfully established a mice POF model induced by D-gal (600 mg/kg/day). We found that after using D-gal, the serum FSH level of mice increased, the E2 level decreased and the total SOD enzyme activity and antioxidant GSH were significantly lower. Moreover, the proportion of primordial follicles was more significantly reduced (Figure 1). Therefore, any approach inhibiting oxidative stress may potentially display the prevention and treatment of POF.

Nrf2 can bind to AREs in the promoter region of the Nrf2 target gene, which can also be an adaptive response to oxidative stress to remove ROS through continuous enzymatic reactions by activating downstream factors of Nrf2, such as GCLC, NQO1 and HO-1. Previous studies have pointed out that aging may reduce the antioxidant capacity of rat ovaries by regulating downstream genes of Nrf2. In our study, D-gal treatment significantly downregulated the expression of Nrf2 and its downstream antioxidant enzymes GCLC, HO-1 and NQO1 (Figure 5), as well as the anti-senescence protein klotho (Figure 4), but significantly upregulated senescence-associated protein P16 expression in the ovary, suggesting that D-gal damaged the antioxidant capacity of the ovary and may be related to POF. To further verify the protective role of Nrf2 in POF, we produced D-gal-induced POF in wild-type and Nrf2^−/−^ mice. As shown in Figure 2, the characteristics of oxidative stress and POF induced by D-gal, such as FSH, E2, GSH and SOD were more significant in Nrf2^−/−^ mice than in wild-type mice. The results above indicated that Nrf2 knockout significantly damaged the ovarian function and antioxidant capacity of POF mice. In the past decade, the NLRP3 inflammasome has been extensively investigated (Kelley et al., 2019). Studies have shown that NLRP3 is a tripartite protein that consists of the scaffold protein, the adaptor protein ASC and caspase-1 (Schroder and Tschopp, 2010). Previous studies have demonstrated that Nrf2 reverses the adjustment of NLRP3 inflammasome activity by inhibiting the priming step (Liu et al., 2017). The decline in the transcription factor Nrf2 could contribute to NLRP3 inflammasome activation during aging (Zhang et al., 2015; Schmidlin et al., 2019). Moreover, inhibition of the NLRP3 inflammasome could prevent ovarian aging. Our results demonstrated that D-gal-induced NLRP3 and senescence-associated protein P16 were much higher in Nrf2^−/−^ mice, while the anti-senescence protein klotho was much lower (Figures 2G–J), suggesting that Nrf2 could be of great value in protection against D-gal-induced POF by inhibiting NLRP3.

Next, we tested the protective effect of Daph on D-gal-induced POF. We found that Daph treatment protected ovarian function and follicular development in POF mice (Figure 3). Moreover, the ovarian senescence-associated protein P16 and the level of oxidative stress were significantly decreased, while the anti-senescence protein klotho was significantly increased (Figure 4). In addition, the expression of Nrf2 and antioxidases such as GCLC, HO-1, NQO1 was increased, and the expression of TXNIP and NLRP3 was significantly decreased (Figure 5), which confirmed that Daph significantly rescued the POF caused by D-gal. And, we summarized the main experimental results by Daph’s effects in Table 1. However, these effects of Daph were weakened or even lost after Nrf2 knockout, and the most obvious were the effects on the expression of TXNIP and NLRP3 (Figure 6 to Figure 8 and Table 2). Therefore, our results suggested that Daph protects against D-gal-induced POF through activating Nrf2 and then inhibiting the NLRP3 inflammasome.

**TABLE 1:** Daph’ effect in WT mice.

Parameters	Control group	D-gal (600 mg/kg/d) group	D-gal + Daph (15 mg/kg/d) group	D-gal + Daph (30 mg/kg/d) group	D-gal + VE (30 mg/kg/d) group
FSH (mIU/ml)	8.382 ± 0.349	13.868 ± 0.314	12.012 ± 0.315	9.686 ± 0.261	9.354 ± 0.262
E2 (pg/ml)	11.814 ± 0.397	8.548 ± 0.300	9.625 ± 0.4.6	10.820 ± 0.460	11.140 ± 0.343
GSH (umol/L)	76.536 ± 1.516	40.946 ± 1.771	63.118 ± 2.041	74.268 ± 2.783	77.300 ± 1.882
SOD (U/mgprot)	23.240 ± 0.774	15.106 ± 0.604	19.134 ± 0.420	21.092 ± 0.515	22.030 ± 0.615
P16 ^a^	0.283 ± 0.012	1.387 ± 0.009	0.963 ± 0.285	0.320 ± 0.010	0.267 ± 0.012
Klotho ^a^	0.690 ± 0.015	0.227 ± 0.009	0.997 ± 0.047	1.557 ± 0.044	1.627 ± 0.044
Nrf2 ^a^	0.581 ± 0.050	0.275 ± 0.040	1.039 ± 0.089	1.444 ± 0.047	—
GCLC ^a^	0.478 ± 0.071	0.222 ± 0.022	0.931 ± 0.051	1.836 ± 0.073	—
NQO1 ^a^	0.653 ± 0.033	0.211 ± 0.034	1.365 ± 0.085	1.573 ± 0.150	—
HO-1 ^a^	1.056 ± 0.050	0.689 ± 0.040	1.427 ± 0.077	1.560 ± 0.052	—
TXNIP (IOD/Aera)	0.057 ± 0.006	0.155 ± 0.008	0.137 ± 0.002	0.074 ± 0.002	0.070 ± 0.003
NLRPE (IOD/Aera)	0.066 ± 0.005	0.174 ± 0.003	0.104 ± 0.005	0.072 ± 0.004	0.076 ± 0.003

a : Relative protein level of β-actin.

**TABLE 2:** Daph’ effect in Nrf2 Knockout mice.

Parameters	Control group	D-gal (600 mg/kg/d) group	D-gal + Daph (15 mg/kg/d) group	D-gal + Daph (30 mg/kg/d) group	D-gal + VE (30 mg/kg/d)Group
FSH (mIU/ml)	11.310 ± 0.481	16.594 ± 0.546	16.062 ± 0.411	15.920 ± 0.341	15.726 ± 0.308
E2 (pg/ml)	9.944 ± 0.265	6.702 ± 0.360	7.018 ± 0.395	6.814 ± 0.462	7.566 ± 0.319
GSH (umol/L)	47.224 ± 3.012	27.190 ± 1.557	28.870 ± 1.960	30.828 ± 1.705	31.542 ± 1.900
SOD (U/mgprot)	16.056 ± 0.447	9.464 ± 0.714	10.224 ± 0.576	10.632 ± 0.539	11.116 ± 0.540
P16 ^a^	0.998 ± 0.252	2.088 ± 0.157	2.034 ± 0.165	2.083 ± 0.251	2.023 ± 0.150
Klotho ^a^	0.316 ± 0.014	0.232 ± 0.002	0.226 ± 0.003	0.232 ± 0.013	0.238 ± 0.014
Nrf2 ^a^	0.138 ± 0.027	0.042 ± 0.005	0.041 ± 0.003	0.045 ± 0.005	—
NQO1 ^a^	0.148 ± 0.030	0.045 ± 0.005	0.042 ± 0.003	0.047 ± 0.006	—
HO-1 ^a^	0.135 ± 0.025	0.042 ± 0.003	0.42 ± 0.002	0.046 ± 0.005	—
TXNIP (IOD/Aera)	0.100 ± 0.006	0.271 ± 0.010	0.271 ± 0.008	0.267 ± 0.010	0.260 ± 0.009
NLRP3 (IOD/Aera)	0.090 ± 0.020	0.284 ± 0.006	0.283 ± 0.012	0.288 ± 0.007	0.290 ± 0.011

a : Relative protein level of β-actin.

## Conclusion

**FIGURE 9:**
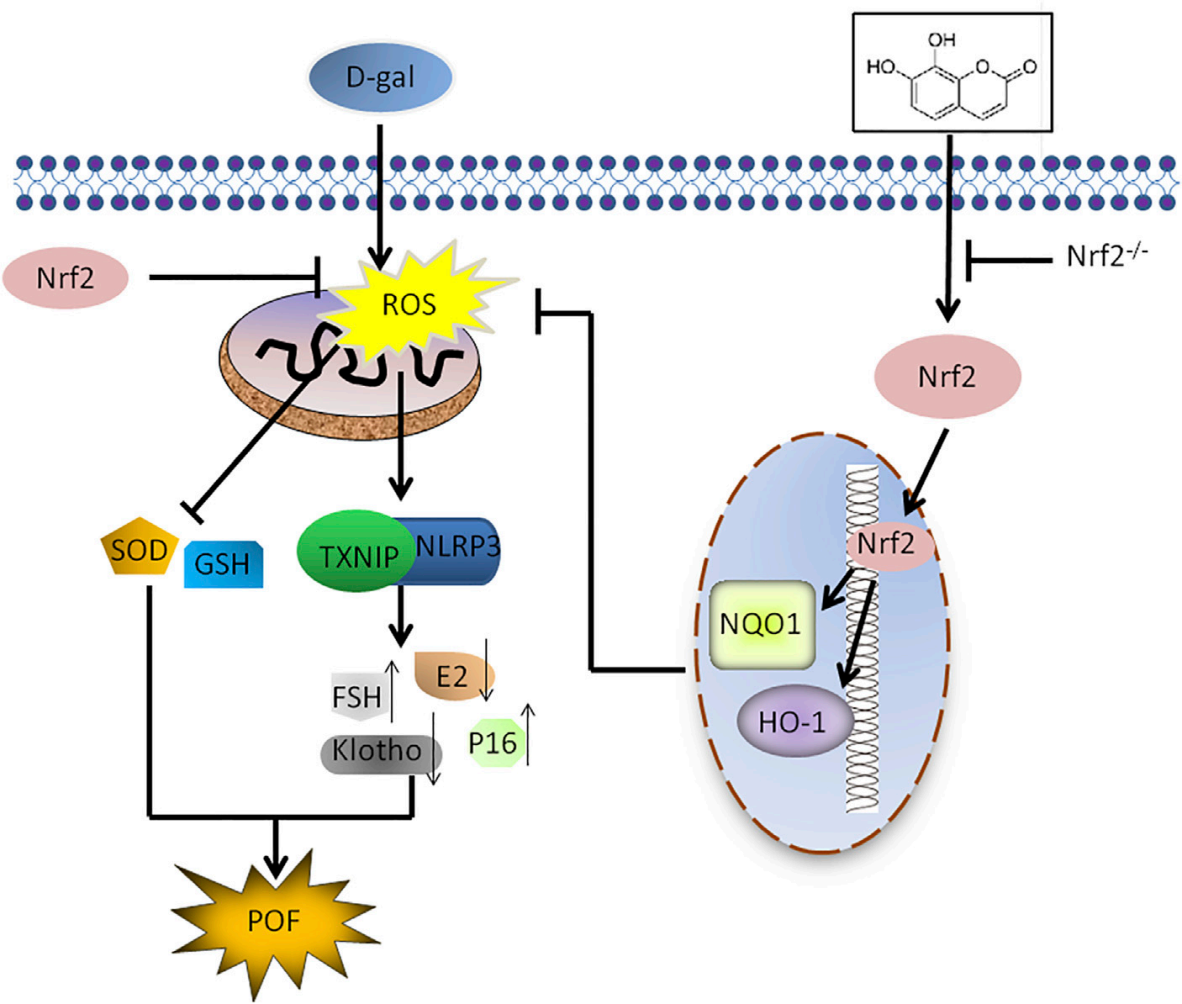
Scheme summarizing the protective effects of Nrf2 activation on D-gal-induced POF *via* inhibiting TXNIP/NLRP3 inflammasome activation. Under D-gal condition, Nrf2 downregulation increases the accumulation of ROS, and then increase TXNIP/NLRP3, p16 and FSH and decrease klotho, SOD, E2 and GSH, leading to POF. Furthermore, Daph inhibits the accumulation of ROS and the activation of TXNIP/NLRP3 inflammsome via activating Nrf2, has a protective effect on D-gal-induced premature ovarian failure.

In summary, we revealed the substantial significance of upregulating Nrf2 to attenuate POF induced by D-Gal. This conclusion stems from three key findings. First, Nrf2 knockout mice were more susceptible to D-Gal-induced POF. Second, the protective effect of daphnetin on D-Gal-induced POF was achieved by activating the antioxidant signaling molecule Nrf2 and inhibiting TXNIP/NLRP3. Finally, treatment of Nrf2 KO mice with daphnetin nearly failed to rescue D-Gal-induced POF. Interestingly, we found the Nrf2/TXNIP/NLRP3 axis may be a promising target in preventing POF (Figure 9). Daph, clinically used to treat rheumatoid arthritis and coagulopathy, is likely to be a candidate drug for the treatment of POF. Further clinical trials are certainly worth investigating the safety and efficacy of daphnetin in POF.

## Data Availability

The original contributions presented in the study are included in the article/Supplementary Material, further inquiries can be directed to the corresponding authors.

## Abbreviations

Daph, Daphnetin; D-gal, D-galactose; FSH, follicle-stimulating hormone insufficiency; GCLC, glutamate cysteine ligase catalytic; HO-1, heme oxygenase-1; KO, knockout; NLRP3, NLR family pyrin domain containing 3; NQO1, NAD(P)H dehydrogenase quinone 1; Nrf2, Nuclear factor erythroid-2 related factor 2; POF, Premature ovarian failure; TXNIP, thioredoxin-interacting protein; VE, Vitamin E; WT, wild-type
